# Site assessment survey to assess the impact of the COVID-19 pandemic on HIV clinic site services and strategies for mitigation in Washington, DC

**DOI:** 10.1186/s12913-023-10069-7

**Published:** 2023-10-20

**Authors:** Nicole Barish, Shannon Barth, Anne K. Monroe, Alan E. Greenberg, Amanda D. Castel, DC Cohort Executive Committee, Natella Rakhmanina, Clover Barnes, Michael Serlin, Princy Kumar, Marinella Temprosa, Vinay Bhandaru, Tsedenia Bezabeh, Nisha Grover, Lisa Mele, Susan Reamer, Alla Sapozhnikova, Greg Strylewicz, Jiayang Xiao, Morgan Byrne, Shannon Hammerlund, Paige Kulie, James Peterson, Bianca Stewart, Yan Ma, Jose Lucar, Jhansi L. Gajjala, Sohail Rana, Michael Horberg, Ricardo Fernandez, Duane Taylor, Jose Bordon, Gebeyehu Teferi, Debra Benator, Rachel Denyer, Maria Elena Ruiz, Stephen Abbott

**Affiliations:** Department of Epidemiology, Milken Institute School of Public Health, George Washington University, 950 New Hampshire Ave NW, 5 th floor, Washington, DC 20052 USA

**Keywords:** HIV, COVID-19, Telehealth, Pandemic, Syndemic, Health services

## Abstract

**Introduction:**

The COVID-19 pandemic has created substantial interruptions in healthcare presenting challenges for people with chronic illnesses to access care and treatment services. We aimed to assess the impact of the pandemic on HIV care delivery by characterizing the pandemic-related impact on HIV clinic-level services and the mitigation strategies that were developed to address them.

**Methods:**

The data comes from a site assessment survey conducted in the DC Cohort, an observational clinical cohort of PWH receiving care at 14 HIV outpatient clinics in Washington, D.C. Frequency counts and prevalence estimates of clinic-level survey responses about the impact of care delivery, COVID-19 testing, and vaccinations and mitigation strategies are presented.

**Results:**

Clinics reported an increase in temporary clinic closures (*n* = 2), reduction in clinic hours (*n* = 5), telehealth utilization (*n* = 10), adoption of multi-month dispensation of antiretroviral (ARV) medication (*n* = 11) and alternative drug delivery via postal/courier service, home/community delivery or pick-up (*n* = 11). Clinics utilized strategies for PWH who were lost to follow-up during the pandemic including offering care to persons with any income level and insurance status (*n* = 9), utilizing e-prescribing for auto refills even if the patient missed visits (*n* = 8), and utilization of the regional health information exchange to check for hospitalizations of PWH lost to follow-up (*n* = 8). Most social services offered before the pandemic remained available during the pandemic; however, some support services were modified.

**Conclusions:**

Our findings demonstrate the extent of pandemic-era disruptions and the use of clinic-level mitigation strategies among urban HIV clinics. These results may help prepare for future pandemic or public health emergencies that disrupt healthcare delivery and access.

**Supplementary Information:**

The online version contains supplementary material available at 10.1186/s12913-023-10069-7.

## Introduction

The COVID-19 pandemic has created substantial interruptions in healthcare, causing many people to be unable to access proper care and treatment for chronic illnesses. It is important to learn from the COVID-19 pandemic to better prepare for future potential disruptions in health care delivery by considering the effect the pandemic has had on the accessibility of clinical care for persons with HIV (PWH) [1]. PWH are vulnerable to disruptions in care and lessons learned can be applied to other risk populations to mitigate detrimental effects of service disruption.

Limited research exists on the relationship between the COVID-19 pandemic and the HIV care continuum and service delivery. A report by the World Health Organization (WHO) estimated that between April and June 2020, 17.7 million people were at risk of ART disruption [2, 3]. One study which reviewed the HIV service delivery literature during the COVID-19 pandemic attributed several factors causing the disruption in HIV care including strict quarantine and lockdown measures (including transportation), shortages of ARVs due to temporary closures of drug manufacturers, and healthcare workers who provide care to PWH redirecting their focus to COVID-19 patients [2]. A survey performed in Eastern and Central Europe found similar results regarding physician care diversion [4].

Another study in New York City assessed the extent of disruption to the HIV care continuum and prevention caused by the COVID-19 pandemic, specifically highlighting the areas of testing, pre-exposure prophylaxis (PrEP), and primary care using community-based organization (CBO) partner-informed research [5]. Structural barriers previously known to be associated with HIV infection were exacerbated by the COVID-19 pandemic, including unemployment, food insecurity, geographic location, difficulty accessing services, and lack of testing and insurance [5]. The study also reported providers at and patients of CBO’s experienced inadequate infrastructure for telehealth, including phone calls due to difficulty navigating the various telehealth platforms, unstable internet access, and limited cell-phone data [5]. These studies highlight the need to further assess the interruptions to HIV care delivery caused by the COVID-19 pandemic and the clinic-level mitigation strategies that were developed to address them. The objective of this study was to assess the impact the pandemic has had on service delivery for people with HIV (PWH). To do so we characterized the pandemic-related impact on HIV clinic-level services and the mitigation strategies developed to address them.

## Methods

A site assessment survey was conducted in the spring of 2022 in the DC Cohort, an observational clinical cohort of PWH at 14 outpatient HIV clinics in Washington, D.C. Previous publications have described the methods of the DC Cohort in detail [6, 7]. Site principal investigators received a one-time electronic questionnaire via REDCap [8] that addressed the impact of the COVID-19 pandemic on the clinic, such as clinic closures, reduction in providers, discontinuation of services, mitigation strategies employed after the pandemic begun, and use of telehealth. Questions for the survey were adapted from several validated questionnaires and reports that investigated the impact of COVID-19 on preparedness and resources, HIV services, telehealth use, and vaccine rollout [9–17]. The survey was reviewed and approved by the GWU Institutional Review Board and determined to be non-human subject research.

Variables included in these analyses fell into the following categories: clinic structure and services offered (medical, social, and laboratory services), reductions in providers, modifications or discontinuation of services offered, and mitigation strategies including ARV delivery, appointment changes, organizational changes/modifications, and strategies for identifying and supporting those lost to follow-up during the pandemic. The survey question “to what degree did COVID-19 and the plans used to manage COVID-19 increase or decrease your clinic's ability to provide the following HIV-related services, compared to pre-pandemic” was assessed over five waves of the pandemic: Wave 1 (March, 2020 to June, 2020 (emerging SARS-CoV-2)), Wave 2 (July, 2020 to September, 2020 (emerging SARS-CoV-2)), Wave 3 (October, 2020 to June, 2021(Alpha and Beta SARS-CoV-2 variants)), Wave 4 (July, 2021 to November, 2021(Delta SARS-CoV-2 variant)), and Wave 5 (December, 2021 to April 2022 (Omicron SARS-CoV-2 variant)). The survey was given at once and the providers were asked to answer the question about each wave at that one time point. This was a cross-sectional one-time survey, that all providers completed at the end of wave five when the entire site assessment survey was sent out. In this analysis data from Wave 1 and Wave 5 are presented to highlight the greatest degree of change. The phrasing of the wave questions remained the same for each wave assessment. For each wave, sites responded about current service delivery compared to the pre-pandemic period. Surveys were sent out electronically between 8 and 18 March. Site PIs were asked to complete the survey on behalf of their clinic. This may have included asking other clinic staff for information to facilitate survey completion; however, they were not required to access patient-level or programmatic level data to complete the survey.

## Statistical analysis

Descriptive statistics were computed for all clinic level factors. Categorical variables were described using frequencies and prevalence estimates. Continuous variables were described using medians and interquartile ranges. Distributions were assessed for departures from normality by examining Q-Q plots, histograms, and the Shapiro–Wilk test. Statistical Analysis Software (SAS, Cary, NC) version 9.4 was used for all analyses [18].

## Results

Table 1 shows descriptive characteristics of the DC Cohort clinics by clinic type, size, number of providers, and COVID testing and vaccine support. All 14 DC Cohort clinic principal investigators responded to the survey. Fifty percent of the clinics were community-based, 50% were hospital-based, and the majority were Ryan White funded clinics (64%).

**Table 1:** Descriptive characteristics of DC cohort HIV clinical sites, Pre and Peri-pandemic (*N* = 14 Clinics)

*Baseline characteristics*	*n* (%)
Clinic Type	
Community-based	7 (50%)
Hospital-based	7 (50%)
Ryan White Clinic	
Yes	9 (64%)
No	5 (36%)
Clinic Size	n
Overall patients cared for at all clinics	16,192
Patients enrolled in DC Cohort	11,469
Use of telehealth^1^ prior to the pandemic	
< 1 year	5 (62.5%)
1–5 years	3 (37.5%)
Number of HIV Care Providers Pre-Pandemic	median (IQR)
HIV clinical providers	5.5 (5–14)
Case managers	2 (1–4)
Eligibility specialists	1 (0–3)
Peer navigators	0.5 (0–2.5)
Community health workers	0 (0–2)
Pharmacists	0 (0–1)
*Pandemic clinic services*	
Clinic closure during the pandemic	
No	12 (85.7%)
Yes	2 (14.3%)
SARS-CoV-2 Testing on Site	
Yes	11 (78.6%)
No	3 (21.4%)
Type of SARS-CoV-2 Testing	
PCR test	11 (78.6%)
Rapid antigen test	7 (50%)
Antibody test	5 (35.7)
Length of time to receive SARS-CoV-2 test results	
2–3 days	7 (63.6%)
Next day	3 (27.3%)
Same day	1 (9.1%)
COVID Vaccine Administration at Clinic	
Yes	11 (78.6%)
No	3 (21.4%)

^1^Telehealth is defined as health care provided electronically and remotely, for the purposes of this study telehealth was defined as any virtual platform, video, phone, or other

Only two clinics reported closing temporarily during the pandemic (Table 1). One clinic reported being closed for four months, in which only telehealth, prescription filling and mailing, drawing labs, and urgent care services were offered to patients, and the other reported that their two locations closed at different periods of time during 2020 for approximately 3 months each, with consolidation of services at the other site and through telehealth. Seventy-nine percent of clinics offered COVID-19 testing, the majority of which were PCR (*n* = 11) and 50% rapid antigen tests (*n* = 7), most providing results within two to three days (*n* = 7). Most clinics provided COVID-19 vaccinations at their clinics (*n* = 11). Two clinics reported referring patients to a specific location for vaccinations, and two reported notifying all patients about where vaccinations were locally offered (data not shown).

Nearly 63% of clinics reported providers using telehealth prior to the pandemic and had been utilizing telehealth for less than one year pre-pandemic (Table 1). When assessing the frequency of providers offering telehealth at each clinic, the majority (*n* = 11) reported that less than ten percent of providers at the clinic utilized telehealth prior to the pandemic, and most (*n* = 10) experienced an increase in the prevalence of providers utilizing telehealth during the pandemic (data not shown). All 14 clinics reported having labs drawn on site pre-pandemic and the majority (*n* = 12) reported continuing this service (data not shown). However, one clinic provider detailed that during the first wave of the pandemic, labs were performed at commercial labs, although the in-house labs were never completely closed as the clinic is a part of a larger hospital organization.

Table 2 presents the frequency counts and percentages of clinic service modifications and strategies for identifying and supporting those lost to follow-up at the clinic sites. Several strategies were adopted throughout the pandemic with respect to ARV access and organizational changes. With respect to ARV access, most clinics adopted multi-month dispensation of ARV medication (*n* = 11) and alternative drug delivery via postal/courier service, home/community delivery, or pick-up (*n* = 11). Regarding appointment strategies, most clinics used staff working at home to contact patients remotely to encourage appointment attendance (*n* = 9). Organizational strategies reported by clinics included reduced clinic hours (*n* = 5). Strategies for identifying and supporting those lost to follow-up during the pandemic included offering care to persons with any income level and insurance status (*n* = 9), using e-prescribing for auto refills even if the patient missed visits (*n* = 8), and checking for hospitalizations of lost patients using the CRISP electronic health record system (*n* = 8).

**Table 2:** Characteristics of service modification at DC Cohort HIV clinical sites throughout the COVID-19 Pandemic (*N* = 14)

Mitigation Strategies *N*(%)	
**ARV Adherence Strategies**	
Multi-month dispensation of ARV medication^a^	11 (78.6%)
Alternative drug delivery (i.e., delivery via postal/courier, home/community delivery, or pick-up)	11 (78.6%)
Use of staff working at home to contact patients remotely to inquire about perceived barriers to regimen maintenance throughout the pandemic	7 (50%)
**Appointment Strategies**	
Use of staff working at home to contact patients remotely to encourage appointment attendance	9 (64.3%)
Provision of appointment reminders to patients with missing viral load measures (w/in 6-month window)	7 (50%)
Prioritization of appointments to patients without viral load measures (w/in 6-month window)	7 (50%)
Prioritization of appointments for those with changes in their health	7 (50%)
Reorganization of appointments, only allowing scheduled visits	6 (42.9%)
Prioritization of appointments for those without HIV/related illness symptoms	2 (14.3%)
**Organizational Changes/Modifications**	
Reduced clinic hours	5 (35.7%)
Mobile clinics to reach patients	2 (14.3%)
Scale up HIV self-testing	2 (14.3%)
Added new staff	2 (14.3%)
Extended clinic hours	1 (7.1%)
Laid off/furloughed staff	0 (0%)
Reduced staff hours	0 (0%)
Reduced Staff salaries	0 (0%)
**Strategies for Identifying and Supporting Those Lost to Follow-Up During the Pandemic**	
Provider offered care to persons with any income level and insurance status^*^	9 (64.3%)
Use e-prescribing for auto refills, even if the patient missed visits^*^	8 (57.1%)
Use CRISP^**^ to check for hospitalizations of lost patients	8 (57.1%)
Systematic monitoring of retention in care (e.g., monitoring visit adherence, gaps in care, or visits per interval of time)^*^	7 (50%)
Provided patients navigation services (accompanying to appointments as needed)^*^	7 (50%)
Check vital records for death certificates of patients lost to follow-up	1 (7.1%)

^*^Strategies in place prior to the pandemic

^**^CRISP Chesapeake Regional Information System for Our Patients is the regional health information exchange

**Fig. 1:**
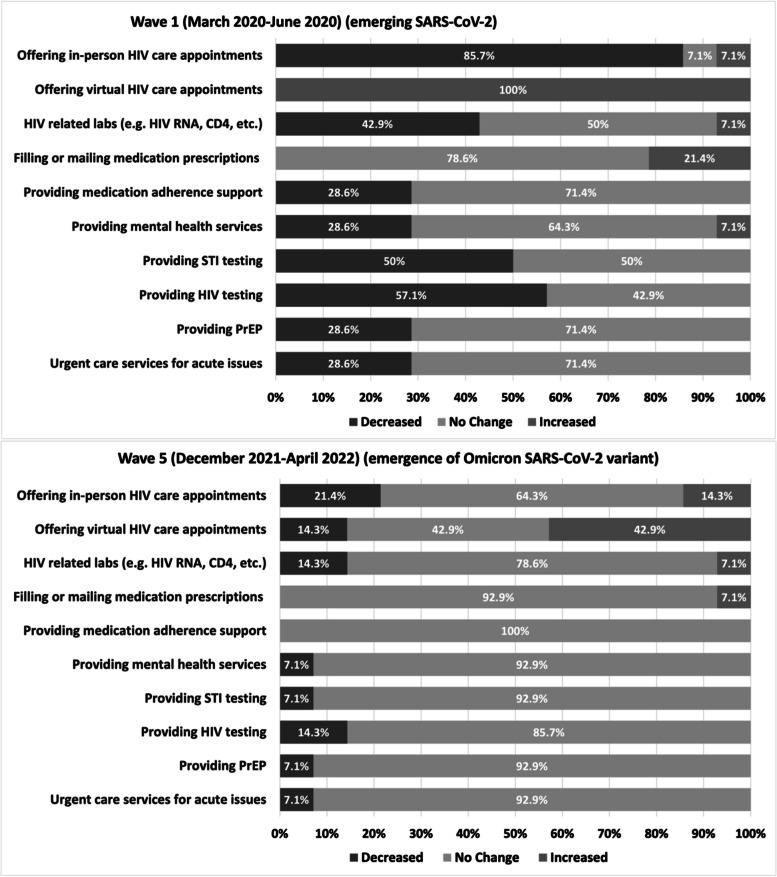
DC Cohort HIV Clinic Service Changes throughout the COVID-19 Pandemic Comparing Wave 1 and Wave 5 Relative to the Pre-Pandemic Era

Figure 1 shows the percentage of clinics that reported a decrease in a particular service in pandemic Wave 1 (March 2020 to June 2020) and pandemic Wave 5 (December 2021 to April 2022) compared to the pre-pandemic era. The services most impacted by the pandemic were in-person HIV care appointments and virtual HIV care appointments. Comparing Wave 1 changes and Wave 5 changes, in-person care observed an 85.7% decrease in Wave 1 and a 21.4% decrease in Wave 5, compared to the pre-pandemic era. Additionally, in Wave 1 virtual care increased by 100%; however, in Wave 5 it was observed to have increased by 42.9%, compared to the pre-pandemic era. All other services offered experienced a decrease in Wave 1 compared to the pre-pandemic era. However, by Wave 5 all other services experienced mostly no change in service availability compared to the pre-pandemic era. Data on service changes in Waves 2, 3 and 4 were also reported (See Supplemental Figure).

There was minimal impact of the pandemic on medical, social, and laboratory services offered: on-site clinical pharmacy, urgent care, job training referrals, substance abuse counseling, opioid treatment programs, nurse navigation, housing referrals, transportation services, and STI testing. Although most services offered before the pandemic continued to be steadily available, some clinics modified services including: on-site clinical pharmacy (*n* = 1), urgent care (*n* = 1), substance abuse counseling (*n* = 2), case management (*n* = 1), and peer intervention programs (*n* = 1). None of these services were permanently discontinued at any of the clinics. Additionally, the site that temporarily closed continued to only offer virtual care and fill/mail prescriptions, while lab draws and urgent care services remained available at the clinic’s hospital location.

## Discussion

The COVID-19 pandemic altered the delivery of healthcare services, requiring providers to identify new ways to safely offer care to patients. Maintaining safe service delivery for PWH has been a crucial goal for HIV clinics during all phases of the COVID pandemic to ensure patients remain engaged in HIV care and simultaneously not put at risk of COVID exposure.

Due to the increased risk of COVID infection in this immunocompromised population, with an estimated 17% self-reported incidence among DC Cohort participants [19], HIV clinicians increased their availability of telehealth offerings. The site assessment survey responses reflect increased flexibility in offering access to patients through virtual means when in-person care was not feasible. This increased the capacity of providers to assist patients who could not physically come into clinic, whether that be due to individual reasons or clinic closures. Recent research has demonstrated that telehealth is a beneficial form of differentiated care delivery, and it should remain a permanent infrastructure to aid in the expansion of care for people with HIV [20].

This analysis found that clinic-level characteristics most affected by the pandemic included temporary clinic closures; reductions in clinical providers, case managers, peer navigators; and modified substance abuse counseling.

This study is subject to several limitations. The survey design itself may introduce bias as the questions may have been interpreted differently by each person who completed the questionnaire. The study presents only observational data, at the site level, and is based on PI self-report data rather than claims or electronic medical record data, therefore self-report bias may be present. Additionally, recall bias may also be present as providers were asked at the end of Wave 5 to reflect on service changes throughout various time points during the pandemic. Lastly, this study is only reflective of the provider perspective and does not seek the perspective of PWH seeking care throughout the pandemic. Ongoing research to characterize PWH perspectives in the DC Cohort is underway [21, 22]. This study also has several strengths. The analysis captures the temporality of the pandemic impact due to the design of pre- and peri-pandemic questions and analyses focused on the various waves of the pandemic. The use of open-ended questions also enabled respondents to clarify their responses.

## Conclusions

Results of this survey highlight the complex nature of intersecting epidemics such as HIV and COVID-19. HIV providers had to alter the way that they had traditionally provided care for patients given pandemic restrictions. While many clinics were able to sustain some core services, our findings demonstrate the extent of pandemic-era disruptions and the use of clinic-level mitigation strategies among urban HIV clinics. Importantly, clinics were able to also ensure continued access to nonmedical services such as mental health, which are often critical wrap around services for PWH. These results emphasize the importance of readily accessible alternative methods of delivery within health systems in preparation for any future shutdowns caused by unprecedented events such as the COVID-19 pandemic. While deploying services such as telehealth, multi-month dispensation, and innovative strategies for engaging people in care were necessary during the pandemic they should become mainstays of HIV care delivery in the post-pandemic era. We recommend that clinics adopt standard protocols for sustaining provision of healthcare and access to social services in emergency situations, in which routine service and delivery may be disrupted.

### Supplementary Information

**Additional file 1: Supplementary Figures: **DC Cohort HIV Clinic Service Changes throughout the COVID-19 Pandemic Waves 2, 3 and 4.

**Additional file 2:** Site Assessment Survey: Provider Survey.

## Data Availability

All data generated or analyzed during this study are included in this published article [and its supplementary information files]. If an outside investigator is interested in obtaining the survey responses, they should reach out to the DC Cohort PI, Dr. Amanda Castel at acastel@gwu.edu, as she will seek DC Cohort Executive Committee approval to share the dataset as outlined in DC Cohort policies and procedures for data access.
