# Supplementary material for: Site assessment survey to assess the impact of the COVID-19 pandemic on HIV clinic site services and strategies for mitigation in Washington, DC
Source: BMC Health Serv Res. 2023 Oct 20;23:1130. doi: 10.1186/s12913-023-10069-7 (PMC10588010; doi:10.1186/s12913-023-10069-7)

| **Supplementary Figure. DC Cohort HIV Clinic Service Changes throughout the COVID-19 Pandemic Waves**  **2, 3 and 4.** |  |
| --- | --- |


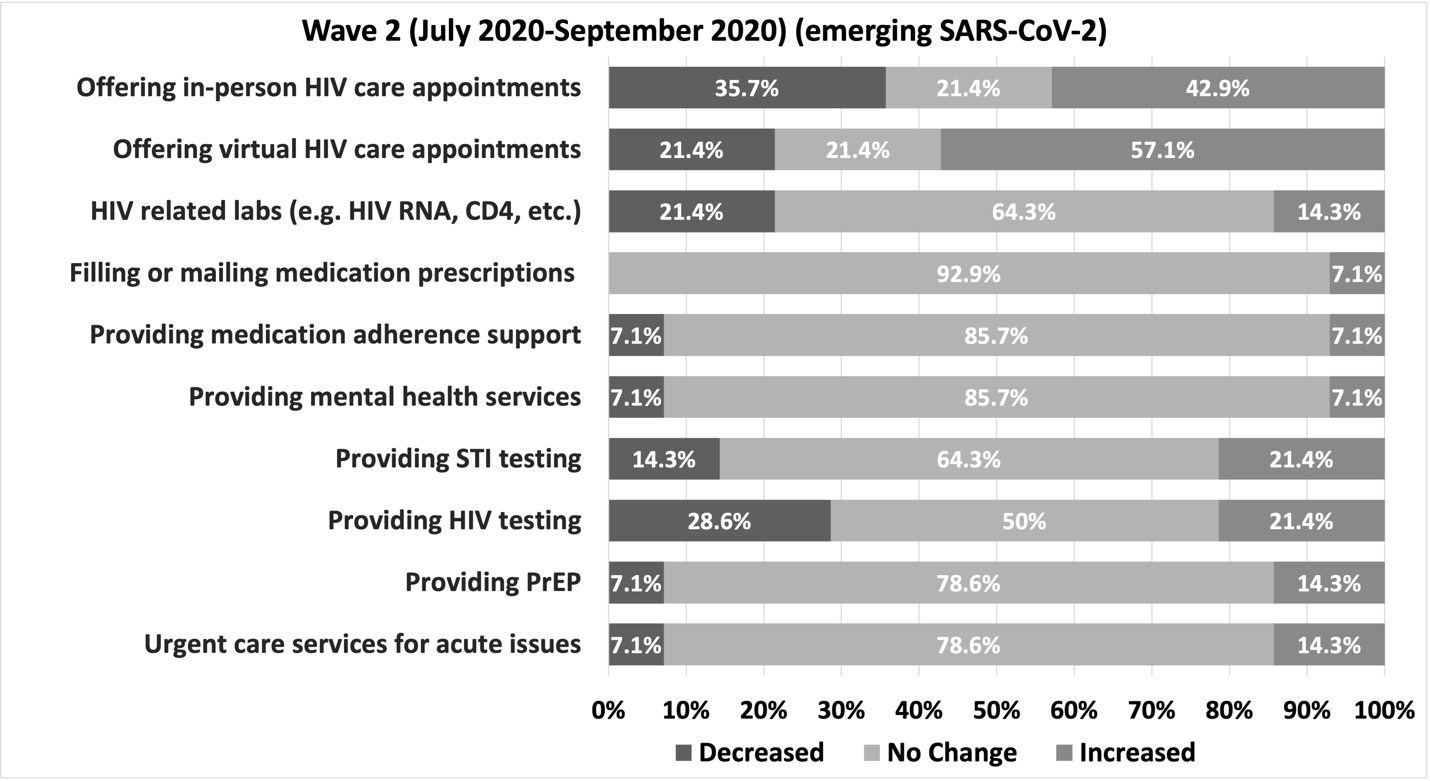


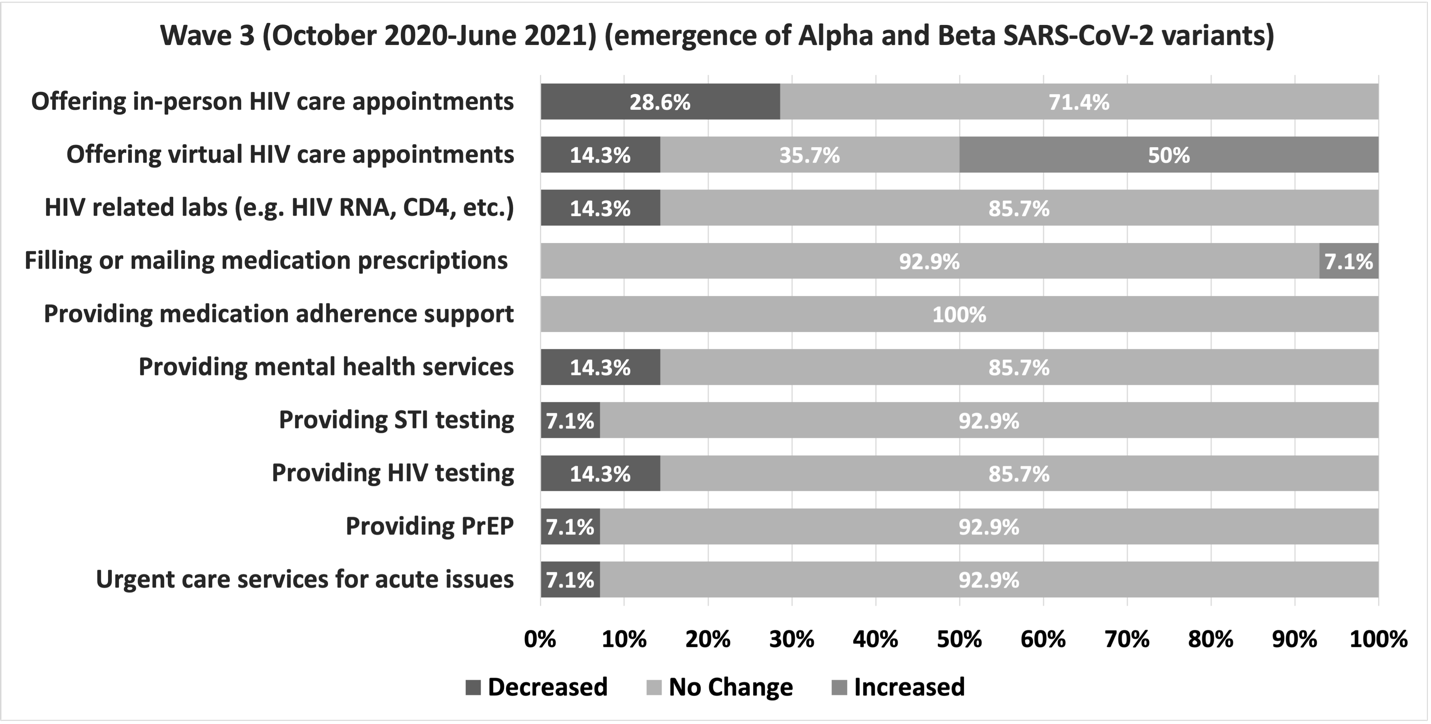


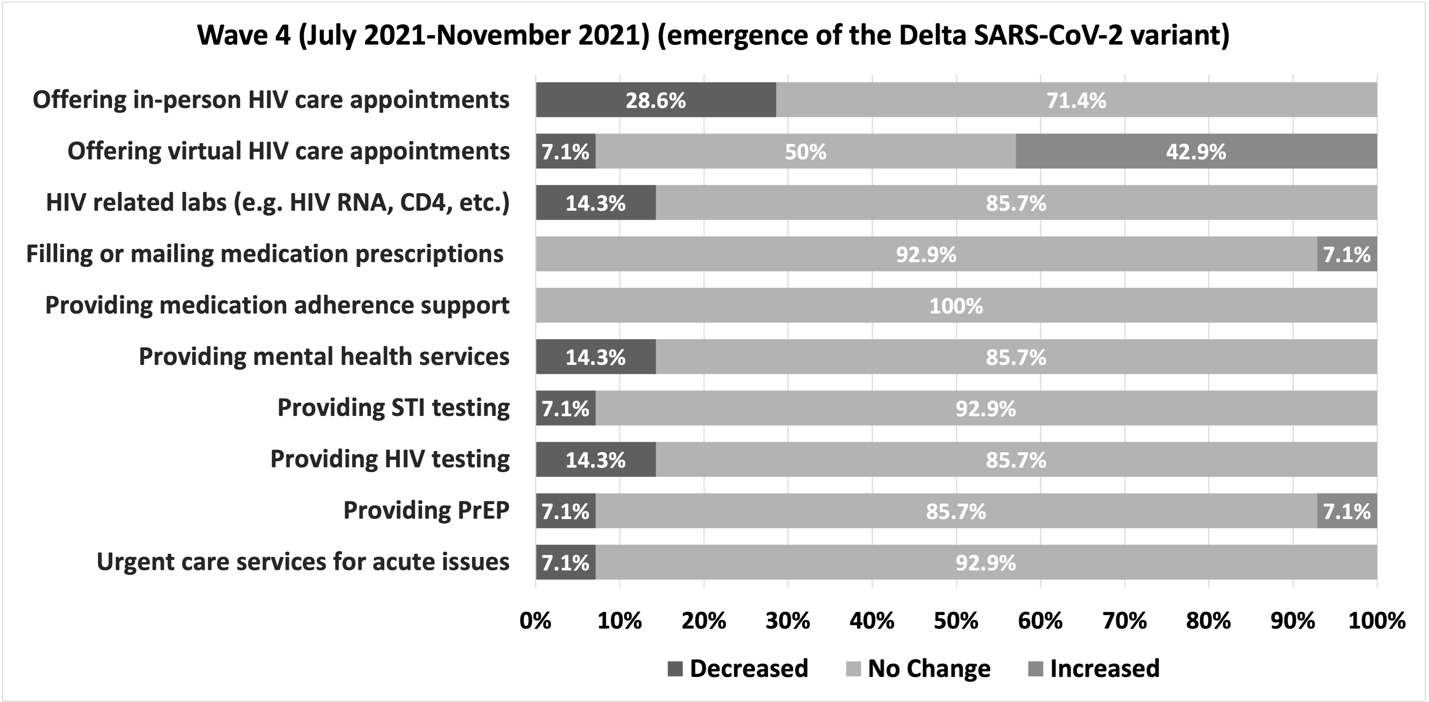

Supplement: Supplementary file 1 — Additional file 1: Supplementary Figures: DC Cohort HIV Clinic Service Changes throughout the COVID-19 Pandemic Waves 2, 3 and 4. [file 12913_2023_10069_MOESM1_ESM.docx]
