# Supplementary material for: Site assessment survey to assess the impact of the COVID-19 pandemic on HIV clinic site services and strategies for mitigation in Washington, DC
Source: BMC Health Serv Res. 2023 Oct 20;23:1130. doi: 10.1186/s12913-023-10069-7 (PMC10588010; doi:10.1186/s12913-023-10069-7)
Supplement: Supplementary file 2 — Additional file 2: Site Assessment Survey: Provider Survey. [file 12913_2023_10069_MOESM2_ESM.pdf]

Provider Survey

Please complete the survey below.

Thank you!

DC Cohort COVID-19 Site Assessment Survey, Washington, DC 2022
Thank you for taking the time to complete this survey. As you well know, the DC Cohort is able to provide robust information on the clinical characteristics of the participants, however, it is also important for us to identify the environment in which care is being delivered.
During the COVID-19 pandemic, clinical care has been disrupted, adapted, and modified in various ways. This survey seeks to systematically characterize some of those changes to better understand the clinical data obtained during that time. To that end, to the extent possible, please complete the following survey to assist us in describing care delivery for PWH in the DC Cohort before and during the COVID-19 pandemic. Please indicate the extent to which the statements below are applicable to your clinic. Please answer on behalf of your clinic as a whole and not based on your individual opinion. Additionally, if you provide HIV care at multiple clinics please answer based on your primary clinical DC Cohort site only. Also, be assured that your responses to this and all questions will be kept private and will be used for research purposes only. We anticipate the survey should only take 15 minutes to complete and are requesting that final surveys be submitted by March 18th.

Clinic Site

- ◯ Veterans Affairs Medical Center
- ◯ Whitman-Walker Institute
- ◯ Georgetown University
- ◯ Washington Hospital Center
- ◯ Unity Health Care
- ◯ Howard University Adult Clinic
- ◯ Howard University Pediatric Clinic
- ◯ Family Medical and Counseling Service
- ◯ Children's National Hospital Pediatric Clinic
- ◯ George Washington Medical Faculty Associates
- ◯ MetroHealth
- ◯ La Clinica Del Pueblo
- ◯ Washington Health Institute
- ◯ Kaiser Permanente Mid-Atlantic States

COVID-19 Testing

Is your HIV clinic providing testing for COVID-19?

- ◯ Yes
- ◯ No
- ◯ Don't Know

If yes, what type of testing is being providing? (check all that apply)

- ☐ Rapid Antigen test
- ☐ PCR tests
- ☐ Antibody tests
- ☐ Other

(If other please specify)

If other, please specify

____

Over the past few weeks, how long has it taken most providers at your clinic to receive COVID-19 PCR test results for your patients?

- ◯ Same day
- ◯ Next day
- ◯ 2-3 days
- ◯ 4-6 days
- ◯ 7+ days
- ◯ Unsure
- ◯ N/A

COVID-19 Impact and Response

We would like to understand the impact of the pandemic on your clinic as well as on care delivery. We recognize that responses to the pandemic have shifted over time so we ask that you think about the different phases and time periods as you respond to some of these questions.

Was there ever a period of time where your clinic had to close temporarily and was unable to offer any services to patients because of the pandemic?

- ◯ Yes
- ◯ No

How long was your clinic closed? ______________________________

How many of the following types of providers did you have at your clinic overall pre-pandemic, regardless of full-time employment? If you do not know please leave the question blank or if n/a write zero.

How many HIV clinical providers did you have at your clinic overall pre-pandemic? ______________________________

How many case managers did you have at your clinic overall pre-pandemic? ______________________________

How many peer navigators did you have at your clinic overall pre-pandemic? ______________________________

How many community health workers did you have at your clinic overall pre-pandemic? ______________________________

How many eligibility specialists did you have at your clinic overall pre-pandemic? ______________________________

How many pharmacists did you have at your clinic overall pre-pandemic? ______________________________

How many of the following types of providers do you have at your clinic overall now, regardless of full-time employment? If you do not know please leave the question blank or if n/a write zero.

How many HIV clinical providers do you have at your clinic overall now? ______________________________

How many case managers do you have at your clinic overall now? ______________________________

How many peer navigators do you have at your clinic overall now? ______________________________

How many community health workers do you have at your clinic overall now? __________________________________

How many eligibility specialists do you have at your clinic overall now? __________________________________

How many pharmacists do you have at your clinic overall now? __________________________________

The table below lists medical, social, and laboratory services. Please indicate below whether your clinic's provision of these services to PWH changed during the pandemic.

	Service not offered before the pandemic	Service offered pre-pandemic	Service offered currently	Service modified because of the pandemic	Service discontinued because of the pandemic
On-site clinical pharmacy	○	○	○	○	○
Urgent care	○	○	○	○	○
Job training referrals	○	○	○	○	○
Substance abuse counseling	○	○	○	○	○
Opioid treatment programs	○	○	○	○	○
Case management	○	○	○	○	○
Peer intervention programs	○	○	○	○	○
Nurse navigation	○	○	○	○	○
Housing referrals	○	○	○	○	○
Transportation services	○	○	○	○	○
STI testing	○	○	○	○	○

Please describe here how these specific services may have been modified during the pandemic. ____

The next series of questions ask about services that your clinic may have provided during the various waves of the pandemic. We have identified 5 different time periods as indicated below and based on local DC data.

During the initial emergency response period (Wave 1: March 2020 - June 2020) (emerging SARS-CoV-2), to what degree did COVID-19 and the plans used to manage COVID-19 increase or decrease your clinic's ability to provide the following HIV-related services, compared to pre-pandemic?

	Decreased	No Change	Increased
Offering in-person HIV care appointments	○	○	○
Offering virtual HIV care appointments (e.g. telehealth)	○	○	○

Drawing HIV related labs (e.g. HIV RNA, CD4, etc.)	◯	◯	◯
Filling or mailing medication prescriptions	◯	◯	◯
Providing medication adherence support	◯	◯	◯
Providing mental health services	◯	◯	◯
Providing STI testing	◯	◯	◯
Providing HIV testing	◯	◯	◯
Providing PrEP	◯	◯	◯
Urgent care services for acute issues	◯	◯	◯

During the second phase of emergency response period (Wave 2 :July 2020 - September 2020) (emerging SARS-CoV-2), to what degree did COVID-19 and the plans used to manage COVID-19 increase or decrease your clinic's ability to provide the following HIV-related services, compared to pre-pandemic?

	Decreased	No Change	Increased
Offering in-person HIV care appointments	◯	◯	◯
Offering virtual HIV care appointments (e.g. telehealth)	◯	◯	◯
Drawing HIV related labs (e.g. HIV RNA, CD4, etc.)	◯	◯	◯
Filling or mailing medication prescriptions	◯	◯	◯
Providing medication adherence support	◯	◯	◯
Providing mental health services	◯	◯	◯
Providing STI testing	◯	◯	◯
Providing HIV testing	◯	◯	◯
Providing PrEP	◯	◯	◯
Urgent care for acute issues	◯	◯	◯

During Wave 3 (October 2020 - June 2021) (emergence of Alpha and Beta SARS-CoV02 variants), to what degree did COVID-19 and the plans used to manage COVID-19 increase or decrease your clinic's ability to provide the following HIV-related services, compared to pre-pandemic?

	Decreased	No Change	Increased
Offering in-person HIV care appointments	◯	◯	◯

Offering virtual HIV care appointments (e.g. telehealth)	◯	◯	◯
Drawing HIV related labs (e.g. HIV RNA, CD4, etc.)	◯	◯	◯
Filling or mailing medication prescriptions	◯	◯	◯
Providing medication adherence support	◯	◯	◯
Providing mental health services	◯	◯	◯
Providing STI testing	◯	◯	◯
Providing HIV testing	◯	◯	◯
Providing PrEP	◯	◯	◯
Urgent care for acute issues	◯	◯	◯

During Wave 4 (July 2021 - November 2021) (emergence of the Delta SARS-CoV-2 variant), to what degree did COVID-19 and the plans used to manage COVID-19 increase or decrease your clinic's ability to provide the following HIV-related services, compared to pre-pandemic?

	Decreased	No Change	Increased
Offering in-person HIV care appointments	◯	◯	◯
Offering virtual HIV care appointments (e.g. telehealth)	◯	◯	◯
Drawing HIV related labs (e.g. HIV RNA, CD4, etc.)	◯	◯	◯
Filling or mailing medication prescriptions	◯	◯	◯
Providing medication adherence support	◯	◯	◯
Providing mental health services	◯	◯	◯
Providing STI testing	◯	◯	◯
Providing HIV testing	◯	◯	◯
Providing PrEP	◯	◯	◯
Urgent care for acute issues	◯	◯	◯

During Wave 5 (December 2021 - present) (emergence of Omicron SARS-CoV-2 variant), to what degree did COVID-19 and the plans used to manage COVID-19 increase or decrease your clinic's ability to provide the following HIV-related services, compared to pre-pandemic?

	Decreased	No Change	Increased
Offering in-person HIV care appointments	◯	◯	◯
Offering virtual HIV care appointments (e.g. telehealth)	◯	◯	◯

Drawing HIV related labs (e.g. HIV RNA, CD4, etc.)	○	○	○
Filling or mailing medication prescriptions	○	○	○
Providing medication adherence support	○	○	○
Providing mental health services	○	○	○
Providing STI testing	○	○	○
Providing HIV testing	○	○	○
Providing PrEP	○	○	○
Urgent care for acute issues	○	○	○

Below is a list of mitigation strategies adopted by other HIV clinics throughout the COVID-19 pandemic. Which of the following, if any, did your clinic utilize? Please select all that apply.

- ☐ Multi-month dispensation of antiretroviral medication
- ☐ Alternative drug delivery: delivery via postal/courier, home delivery or pick-up, and/or community delivery
- ☐ Extended clinic hours
- ☐ Reduced clinic hours
- ☐ Mobile clinics to reach patients
- ☐ Increased PPE in clinic
- ☐ Reorganization of appointments, only allowing scheduled visits
- ☐ Use of staff working at home to contact patients remotely to encourage appointment attendance
- ☐ Use of staff working at home to contact patients remotely to inquire about perceived barriers to regimen maintenance throughout the pandemic
- ☐ Scale up HIV self-testing
- ☐ Prioritization of appointments for those without viral load measures within the past 6 months
- ☐ Provision of appointment reminders to patients with missing viral load measures (within a 6-month window)
- ☐ Prioritization of appointments for those with changes in their health
- ☐ Prioritization of appointments for those without symptoms
- ☐ Added new staff
- ☐ Laid off/furloughed staff
- ☐ Reduced staff hours
- ☐ Reduced staff salaries
- ☐ None of the above

Did your clinic use any of the following specific strategies to track and support people lost to follow up during the pandemic?	☐ Systematic monitoring of retention in care (e.g., monitoring visit adherence, gaps in care, or visits per interval of time)<br>☐ Follow-up with patients who missed appointments via email, phone or some other form of communication<br>☐ Provider offered care to persons with any income level and insurance status<br>☐ Continued case management<br>☐ Provided patient navigation services (e.g., accompanying to appointments as needed)<br>☐ Use e-prescribing for auto refills, even if the patient missed visits<br>☐ Provide prescriptions for longer periods of time (e.g., multi-month dispensing)<br>☐ Use CRISP to check for hospitalizations of lost patients<br>☐ Check vital records for death certificates of lost patients
How were most labs at your clinic drawn at your site before the pandemic?	◯ On site/at our in-house laboratory<br>◯ Lab order sent/Referred elsewhere<br>◯ Not done/postponed<br>◯ Other
If other please specify	__________________________________
How have most labs at your clinic been drawn at your site during the pandemic?	◯ On site/at our in-house laboratory<br>◯ Lab order sent/Referred elsewhere<br>◯ Other
If other please specify	__________________________________

Telehealth

Now we would like to ask you a few questions about your clinic's use of telehealth during the pandemic. Telehealth includes any care provided to a patient that did not occur in person (i.e. telephone as well).

What telehealth platforms was your clinic using prior to the pandemic? (select all that apply)	☐ None<br>☐ Doximity<br>☐ Whats App<br>☐ clinic's EHR<br>☐ Zoom<br>☐ Telephone<br>☐ Other
If other please specify	__________________________________
How long had your clinic been using telehealth to provide patient care prior to the pandemic?	◯ < 1 year<br>◯ 1-5 years<br>◯ 6-10 years<br>◯ >10 years

Does your clinic use any other mobile Health apps, websites, or electronic tools separate from the electronic medical record system to support patient care? (e.g. tools to assist with care coordination, sharing lab results or documents related to care, arrange opportunities for peer support, provider communication with patients or other providers)	◯ Yes<br>◯ No<br>◯ Don't Know
Please specify the name of the tool(s)(e.g. Facetime, Whatsapp, doodle polls, etc)	__________________________________
Please specify the functionality of the tool(s) (e.g., tools to share labs with patients electronically, calendar tools to share appointment info across staff, doodle polls to coordinate patient group meetings, etc.)	__________________________________
Please describe how you access this tool(s) (e.g. website, smartphone app)	__________________________________
Please estimate what percent of providers at your clinic utilized telehealth before the pandemic.	◯ < 10%<br>◯ 10%-24%<br>◯ 25%-49%<br>◯ 50%-75%<br>◯ >75%
Please estimate what percent of providers at your clinic are now utilizing telehealth during the pandemic.	◯ < 10%<br>◯ 10%-24%<br>◯ 25%-49%<br>◯ 50%-75%<br>◯ >75%

For each criteria listed below, did your clinic prioritize patients with HIV for in-person visits or telehealth visits?

	In-Person	Telehealth	There was no clinic-wide standard; individual providers decided	Protocol did not change, patients were not prioritized
Newly Diagnosed	◯	◯	◯	◯
VL>200 copies/mL	◯	◯	◯	◯
CD4< 200 cells/uL or other AIDS-defining illness	◯	◯	◯	◯
Patients with acute/urgent medical conditions	◯	◯	◯	◯
Symptomatic patients	◯	◯	◯	◯
Other	◯	◯	◯	◯

If other please specify	____________________________

How effective has your clinic been in addressing the following aspects of the HIV care continuum using telehealth?

	Very effective	Somewhat effective	Neutral	Not too Effective	Not at all Effective
Providing care to patients with HIV during the pandemic	◯	◯	◯	◯	◯
Providing care to patients with HIV after the pandemic	◯	◯	◯	◯	◯
Retention in care for their patients with HIV	◯	◯	◯	◯	◯
Adherence to medication/maintaining viral suppression for their patients with HIV	◯	◯	◯	◯	◯

Which of the following, if any, does your clinic perceive as barriers to patients with HIV accessing telehealth? Please select all that apply.

- ☐ Lack of patient access to video technology
- ☐ Lack of digital literacy in patients
- ☐ Lack of patient access to internet
- ☐ Patient preference for in-person visits
- ☐ Lack of patient access to data
- ☐ Lack of patient awareness/understanding of telehealth offerings
- ☐ Lack of health insurance
- ☐ Lack of access to community-based resources
- ☐ Concerned about lack of privacy/confidentiality
- ☐ Provider preference (i.e. provider does not utilize telehealth)
- ☐ Other
- ☐ None

If other please specify

____

Does your clinic intend provide care via telemedicine once this pandemic has ended?

- ◯ No, not at all
- ◯ Yes - in some situations / with certain patients
- ◯ Yes - for all situations / patients
- ◯ Unsure

Please indicate the extent to which the statements below are applicable to your clinic.

	Strongly agree	Agree	Neither agree nor disagree	Disagree	Strongly disagree
The use of telehealth has impacted the patient provider relationship in a negative way	◯	◯	◯	◯	◯
The use of telehealth has made it more difficult to accurately diagnosis patients' conditions	◯	◯	◯	◯	◯

The use of telehealth has disrupted the HIV care continuum and service delivery	○	○	○	○	○

Vaccine Rollout

Now we would like to ask you a few questions about COVID-19 vaccine rollout at your clinic.

Has your HIV clinic been administering COVID-19 vaccines to patients with HIV?

- ○ Yes
- ○ No
- ○ Don't Know

If your clinic is not currently administering COVID-19 vaccines, how is your clinic supporting vaccine efforts for patients with HIV? Please select all that apply.

- ☐ Referring patients to a known source when they call to ask for the vaccine
- ☐ Having educational information in your practice to share with patients
- ☐ Proactively notifying all their patients about how to get the vaccine
- ☐ Other

If other please specify

____

Which of the following, if any, does your clinic perceive as barriers to COVID-19 vaccination among patients with HIV? Please select all that apply.

- ☐ Concern about side effects
- ☐ Want to know more about how well it works
- ☐ Don't think they need it
- ☐ Concern about cost
- ☐ Concern that the vaccine was developed too quickly
- ☐ Concern that the vaccine could give them COVID
- ☐ Plan to wait and see if it is safe and may get it later
- ☐ Don't trust the government
- ☐ Plan to use masks/other precautions instead
- ☐ Don't think it will protect them against new variants
- ☐ Some have had COVID already and think they are immune
- ☐ Access to the vaccine
- ☐ Other

If other please describe

____

This is the end of the survey. Thank you very much for your participation. This information will be very useful as we characterize your clinic's responses to the COVID-19 pandemic among DC Cohort clinical sites.
